# *My Migraine Voice* survey: a global study of disease burden among individuals with migraine for whom preventive treatments have failed

**DOI:** 10.1186/s10194-018-0946-z

**Published:** 2018-11-27

**Authors:** Paolo Martelletti, Todd J. Schwedt, Michel Lanteri-Minet, Rebeca Quintana, Veruska Carboni, Hans-Christoph Diener, Elena Ruiz de la Torre, Audrey Craven, Annette Vangaa Rasmussen, Simon Evans, Annik K. Laflamme, Rachel Fink, Donna Walsh, Paula Dumas, Pamela Vo

**Affiliations:** 1 European Headache Federation, Rome, Italy; 2 Department of Clinical and Molecular Medicine, Sapienza University of Rome, Rome, Italy; 3 Department of Neurology, Mayo Clinic, 5777 East Mayo Boulevard, Phoenix, AZ 85054 USA; 4 Département d’Evaluation et Traitement de la Douleur CHU Nice, FHU InovPain, Université Côte Azur, Nice, France; 5 GfK EMER, Madrid, Spain; 6 IPSOS Switzerland (formerly GfK Health), Basel, Switzerland; 7 Department of Neurology and Headache Center, University of Duisburg-Essen, Duisburg, Germany; 8 European Migraine and Headache Alliance (EMHA), Brussels, Belgium; 9 Migraine Association of Ireland (MAI), Dublin, Ireland; 10 Danish Headache Center, Rigshospitalet Glostrup, Copenhagen, Denmark; 11 Migraine Action, Leicester, UK; 12 Novartis Pharma AG, Basel, Switzerland; 13 European Federation of Neurological Associations, Brussels, Belgium; 14 Migraine Again, Irvine, USA

**Keywords:** Migraine, Burden, Global survey, Work productivity, Migraine experience

## Abstract

**Background:**

Migraine is associated with many debilitating symptoms that affect daily functioning. *My Migraine Voice* is a large global cross-sectional study aimed at understanding the full burden and impact of migraine directly from patients suffering from ≥4 monthly migraine days (MMDs) with a history of prophylactic treatment failure.

**Methods:**

This study was conducted worldwide (31 countries across North and South Americas, Europe, the Middle East and Northern Africa, and the Asia-Pacific region) using an online survey administered to adults with migraine who reported ≥4 MMDs in the 3 months preceding survey administration, with pre-specified criteria of 90% having used preventive migraine treatment (80% with history of ≥1 treatment failure). Prophylactic treatment failure was defined as a reported change in preventive medication by individuals with migraine for any reason, at least once.

**Results:**

In total, 11,266 individuals participated in the survey. Seventy-four percent of the participants reported spending time in darkness/isolation due to migraine (average: 19 h/month). While 85% of all respondents reported negative aspects of living with migraine (feeling helpless, depressed, not understood), sleeping difficulties (83%), and fear of the next attack (55%), 57% shared ≥1 positive aspect (learning to cope, becoming a stronger person). Forty-nine percent reported feeling limited in daily activities throughout all migraine phases. Migraine impact on professional, private, or social domains was reported by 87% of respondents (51% in all domains). In the previous 12 months, 38% of respondents had visited the emergency department (average: 3.3 visits), whereas 23% stayed in hospital overnight (average: 3.2 nights) due to migraine.

**Conclusions:**

The burden of migraine is substantial among this cohort of individuals with at least 4 migraine days per month and for whom at least 1 preventive migraine treatment had failed. Interestingly, respondents reported some positive aspects in their migraine journey; the greater resilience and strength brought on by coping with migraine suggests that if future treatments could address existing unmet needs, these individuals with migraine will be able to maximize their contribution to society.

## Background

Migraine is a painful, debilitating neurological disease, which consistently ranks among the top 10 leading causes of years lived with disability (YLD) worldwide [1, 2]. It is currently the first-leading cause of YLD among individuals under 50 years old [3].

The impact of migraine on all domains of life contributes to the complexity of adequately capturing its true wide-ranging burden from the perspective of people with migraine, particularly those experiencing a relatively high frequency of migraine attacks and with unmet treatment needs. Multiple previous studies and surveys described various manifestations of the burden of migraine, such as the American Migraine Prevalence and Prevention (AMPP) study, the International Burden of Migraine Study (IBMS), the Chronic Migraine Epidemiology and Outcomes (CaMEO) study, the Eurolight project, and the Global Burden of Disease (GBD) study [4–10]. However, the current understanding of migraine burden among individuals with the highest unmet needs, specifically those experiencing ≥4 monthly migraine days (MMDs) and prior prophylaxis failure, is limited. This particular subgroup is of interest since new preventive migraine therapies have demonstrated efficacy and safety in this population. Furthermore, understanding the burden of disease in individuals with ≥4 MMDs is very important to enable physicians and/or others involved in migraine management to make well-informed decisions on appropriate preventive migraine care for these people. Specifically, no detailed assessment of the current treatment pathway, impact on work productivity, or burden associated with premonitory and postdromal phases of migraine is available in individuals experiencing ≥4 MMDs who have had previous prophylactic treatments that failed and continue to experience frequent migraines.

A qualitative study was conducted using online bulletin boards (OBBs) to identify the key issues experienced by people living with migraine prior to the present global study [11, 12]. OBBs are a qualitative data collection method that allow anonymous participation across varied geographic locations, customization of questions, and interactions and answers in local languages to facilitate and enhance data quality; qualitative data from OBBs were used to inform the *My Migraine Voice* study. The OBB study highlighted the challenges and important impacts of migraine on many aspects of the daily lives of individuals with migraine. This permitted the substantial functional and emotional burden associated with migraine to be described by affected individuals in their own words [11, 12]. People with migraine reported facing significant challenges in pursuing daily activities or work and difficulties with coping mechanisms. The negative impact of migraine also extended to caregivers, most of them being torn between commitment, self-sacrifice, and resentment in relation to their responsibilities toward the person with migraine for whom they were providing care [11, 12].

Building on this first initiative, the quantitative *My Migraine Voice* study was undertaken on a much larger and comprehensive scale via an online survey to further understand views of people with migraine and its worldwide impact across 31 countries. The objectives of *My Migraine Voice* were to assess migraine characteristics and describe the current real-world burden and impact of living with migraine from clinical, personal, and economic perspectives among adults with migraine experiencing ≥4 MMDs.

## Methods

### Study design and participants

This was a large, cross-sectional, multi-country online survey of adult participants (≥18 years of age) with migraine. Screening questions to determine eligibility included a description of migraine based on the International Classification of Headache Disorders 3rd edition (ICHD-3) criteria followed by a series of migraine symptom and characteristic questions to qualify that participants were experiencing migraine. Participants could also record whether they had received a medical diagnosis of migraine, although this was not part of the inclusion criteria. Further, only those who self-reported experiencing ≥4 MMDs each month for the previous 3 months were eligible for inclusion in the survey.

Prespecified quotas were applied to people reporting a history of taking a prophylactic medication to prevent their migraine: 90% of participants reported current or previous use of preventive migraine medication, of which 80% switched preventive treatment, and the remaining 10% were preventive treatment-naïve. Individuals with migraine who reported changing their preventive medication for any reason at least once were defined to have had a preventive treatment failure (TF). The applied quotas were required to ensure that the occurrence of TF was adequately represented among study participants, with a substantive proportion of individuals that had a history of ≥2 TFs.

Participants were recruited by means of existing online panels (GfK Health) and support organizations for people with migraine. Participants’ consent was obtained prior to participation in the survey and those who completed the survey were compensated with an incentive in the form of a voucher and aligned with local standards. Data were collected via Confirmit, an online platform, and internet surveys were completed independently by respondents. Data were handled confidentially and anonymity of respondents was maintained throughout the study. As such, and due to its research format, this study was exempted from ethics committee review.

### Survey design and outcomes

The survey drew upon the previous OBB study results. Details on the methods of the OBB study are published elsewhere [11, 12]. A steering committee composed of migraine specialists, a specialized nurse, and patient support group leaders also informed the methodology and additional topics of importance. The final survey comprised 88 questions and included country-specific questions tailored to reflect differences in healthcare systems and available treatments.

Table 1 presents the outcome parameters assessed in the survey relating to sociodemographic factors, health and medical history, health-related quality of life, impact on daily activities, treatment patterns and participants’ treatment experience, and healthcare utilization.

**Table 1:** Parameters Collected in the Survey

Domain	Items
Sociodemographic characteristics	• Age • Gender • Income • Place of living • Occupation status • Family status
Health/medical history	• Monthly migraine days • Monthly headache days • Characteristics of migraine (eg, symptoms, pain, nausea, aura, duration) • Medication history • Migraine history (eg, time affected by migraine, diagnosis, time since diagnosis, engagement with HCPs) • Family history of migraine and headache (parents, siblings, children) • Comorbidities (chronic conditions)
QoL	• QoL • Experience of living with migraine (eg, fear of migraine attack, ability to focus, fatigue, feelings of frustration/hopelessness)
Healthcare utilization	• Healthcare utilization (eg, hospitalizations, ED visits, outpatient visits, general practitioners, neurologists, headache centers, brain scans) • Treatments for relief of headache/migraine • Treatments for prevention of headache/migraine • Non-pharmacological management
Impact on daily life	• Work status (including changes in employment due to migraine) • Daily activities and household activities • Work productivity using WPAI-Migraine
Treatment patterns/ participants’ treatment experience	• Time on treatment • Treatment satisfaction • Reason for switching (if any)

*ED* Emergency department; *HCP* Healthcare provider; *QoL* Quality of life; *WPAI* Work Productivity and Activity Impairment

Because of limited published information on the burden before and after the migraine attack, the survey included questions on the migraine phases. Descriptions of migraine phases were provided to participants (Table 2).

**Table 2:** Description of Migraine Phases

Premonitory Phase (Before the Attack)		Headache Phase (The Attack)	Postdromal Phase (After the Attack)	
Warning phase *Might occur days or hours before* • Concentration problems • Irritability • Repetitive yawning • Sleep issues • Food cravings • Tiredness	Aura • Sensory disturbances • Speech disturbances • Visual disturbances • Symptoms last up to one hour	The attack *(and accompanying symptoms)* • Throbbing one-sided headache • Sickness, nausea • Sensitive to light, sound	Resolution • Sleep helps • Fatigue • Food intolerance • Altered mood • Impaired concentration • Less intense pain	Recovery • Hangover feeling • Weak • Need rest

In addition, a validated questionnaire was included in the survey to assess the impact of migraine on work productivity and daily activities among employed respondents. The impact of migraine on work productivity and regular activities during the past 7 days was evaluated using the Work Productivity and Activity Impairment (WPAI) questionnaire and was compared among treatment -naive, no prior TF, 1 TF, and ≥ 2 TF patient subgroups.

### Data analysis

Data analysis was conducted using IBM SPSS Statistics (version 24) to generate summary statistics on country and aggregate levels, and Qlikview software was used for charting. Quality assessment of the data was performed and included an evaluation of response patterns and inconsistencies and an analysis of answers to open-ended questions. Comparisons of different subset of participants were completed using frequencies, means, medians, and standard deviations. The reported *P*-values are from the T-student test when testing differences between averages, and from the Chi-square test when testing differences between percentages or proportions.

## Results

**Fig. 1:**
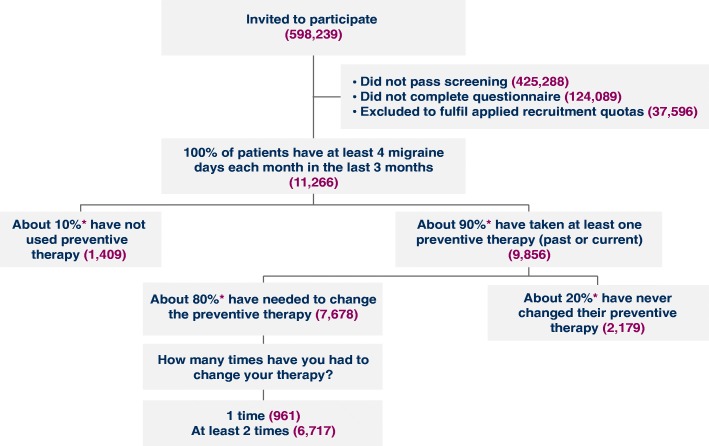
The *My Migraine Voice* Study Population Accrued per Applied Criteria. *Predetermined quotas

Worldwide recruitment occurred from September 2017 to February 2018 across 31 countries in North America (*n* = 1689) and South America (*n* = 1438), Europe (*n* = 6156), the Middle East and Northern Africa (*n* = 1111), and the Asia-Pacific region (*n* = 872), resulting in the participation of a total of 11,266 individuals with migraine in *My Migraine Voice* (Fig. 1). Per the predefined inclusion criteria, approximately 90% of participants (*n* = 9856) had taken preventive medication (past or present) for migraine, and ≥ 80% of these (*n* = 7678) had experienced at least 1 or more TF, which represents 68% of the total survey population (Fig. 1). The majority of participants with a previous treatment failure were those who had ≥2 TFs (*n* = 6717; 87% of those with any TF).

The characteristics of the study participants are summarized in Table 3. Of the total 11,266 participants, a majority were female, had children, and had a family history of migraine. The majority of participants were in either full- (47%) or part-time (12%) paid employment or self-employment (7%) or were students (7%). On average, participants had migraine for 11.6 years, but over one-quarter (27%) had it for more than 20 years. The majority of participants (86%) also reported currently having a healthcare professional for their migraine, most frequently a neurologist or headache specialist (41%) or general practitioner (42%).

**Table 3:** Baseline Characteristics of the *My Migraine Voice* Population

	Total Population (n = 11,266)	Migraine Individuals With 1 TF (*n* = 961)	Migraine Individuals With ≥2 TFs (n = 6717)
Gender			
Female	75% (8396)	77% (735)	73% (1859)
Male	25% (2689)	24% (226)	27% (652)
Age (mean)	39.4 years	39 years	40.2 years
With children (yes)	63% (7098)	58% (557)	67% (4500)
Employed (full time or part-time)	58% (6534)	57% (548)	59% (3963)
Family history of migraine (yes)	54% (6083)	53% (509)	56% (3761)
Time being affected by migraine (mean)	11.6 years	10.9 years	12.2 years
< 1 year	4% (423)	5% (49)	2% (150)
1–5 years	26% (2929)	29% (281)	23% (1537)
6–10 years	21% (2381)	21% (199)	22% (1492)
11–15 years	12% (1350)	10% (96)	13% (852)
16–20 years	10% (1149)	9% (84)	11% (717)
21 years or more	27% (3034)	26% (252)	29% (1969)
Taking acute migraine medications	76% (8562)	75% (721)	81% (5440)
Average number of self-reported chronic conditions	3.3	3.1	3.4
First most commonly reported: anxiety	27% (3042)	25% (240)	28% (1881)
Second most commonly reported: insomnia/ sleep disorder	24% (2704)	21% (202)	26% (1746)
Third most commonly reported: depression	23% (2591)	22% (211)	25% (1679)

*TF* Treatment failure

Seventy-six percent of survey participants reported taking medications for acute treatment, which increased to 81% among those with ≥2 TFs. Of the participants taking medications for acute treatment, 83% took these based on a doctor’s prescription and 51% also took over-the-counter (OTC) medications, indicating that a substantive proportion of respondents were taking both doctor-prescribed and OTC medications. Notably, among respondents taking acute treatments, 20% reported using opioids for their migraine. Overall, study respondents reported having 3.3 other chronic conditions on average, with anxiety, insomnia/sleep disorders, and depression being most commonly reported in all participants, irrespective of whether they had experienced previous preventive TF.

### Migraine characteristics

Considering all phases of the migraine attack, survey participants had on average 9.8 days/month affected by migraine over the last 3 months. For 44% of respondents, migraine episodes lasted 1 to 2 days or more, and 19% reported episodes lasting longer than 3 days. A headache phase duration longer than 1 day was reported by 27% of respondents without any TF compared with 38% of respondents who had ≥2 TFs (*P* < 0.05) (Table 4). Almost half of respondents (49%) reported feeling limited in completing daily activities throughout the 3 migraine attack phases (feeling limited over a total of 10.5 days/month on average). Peak of limitations occurred during the headache phase as reported by 71% of respondents vs 75% for those with ≥2 TFs (*P* < 0.05) who felt very to extremely limited during the phase. About one-third of respondents also felt this level of limitation in the premonitory and postdromal phases (Table 4). Very few respondents reported feeling “no limitation” in each of the migraine phases (4%, 1%, and 5% in the premonitory, headache, and postdromal phases, respectively).

**Table 4:** Characteristics of Migraine Phases

	Premonitory Phase, % (N)	Headache Phase, % (N)	Postdromal Phase, % (N)
Duration of phase			
< 4 h	50% (5676)	20% (2280)	30% (3419)
4–24 h	30% (3312)	45% (5067)	40% (4516)
> 24 h	14% (1622)	34% (3835)	27% (3006)
Not experiencing this phase	6% (656)	1% (84)	3% (325)
Proportion of individuals in each preventive treatment group reporting the duration of this migraine attack phase > 24 h:			
- No failure to migraine preventive treatment	11% (250)	27% (599)	20% (436)
- 1 TF of migraine preventive treatment	11% (103)	29% (282)	23% (218)
- ≥2 TFs of migraine preventive treatment	17% (1114)	38% (2539)	31% (2048)
- *P*-value: ≥2 failures vs no failure of migraine preventative treatment	< 0.05	< 0.05	< 0.05
Feeling very to extremely limited during the phase	29% (3047)	71% (8023)	30% (3291)

*TF* Treatment failure

### Living with migraine

Although the main symptom of migraine reported by 86% of respondents was a long-lasting headache (from 4 to 72 h), other symptoms such as severe pain either on one side of the head (75%) or throbbing pain (78%), light sensitivity (80%), sound sensitivity (75%), and nausea (74%) were also reported by 74% to 80% of participants. On a scale from 0 to 10 (highest level), the severity of pain experienced during the last month by respondents was 7.4 on average, including 57% of respondents with a higher than average pain severity (8–10). Notably, overall, 74% of respondents (78% of those with ≥2 TFs) reported spending long periods of time in darkness and isolation—an average of 19 h per month and increasing to 21 h per month in those with ≥2 TFs. Migraine was also reported to cause sleeplessness by 83% of all respondents on average (86% for those with ≥2 TFs).

Table 5 summarizes the range of circumstances and ways in which respondents reported that over the previous 1 month, migraine had negatively affected their daily functioning. Notably, in each case, a higher proportion of respondents with ≥2 TFs reported a negative impact on daily functioning compared with the overall participant population. Four out of 5 respondents reported having to cancel plans due to migraine. When asked how they felt about living with migraine, overall 85% of respondents (88% of those with ≥2 TFs) reported at least 1 negative aspect of living with migraine. The most mentioned aspect was the feeling of being misunderstood by people (48%), followed by depression (41%) and feelings related to hating their own life (39%), feeling helpless (39%), and feeling that migraine controls and dictates their lives (39%). An average of 27% of all respondents were either very or extremely fearful of experiencing another episode of migraine (average of 32% among those with ≥2 TFs). Despite all the negative aspects, 57% of respondents mentioned at least 1 positive aspect of living with migraine, mainly related to gaining resilience and strength: ‘I have learnt to cope with the disease’ (40%), ‘I am responsible for my migraine’ (13%), and ‘It has made me stronger’ (11%).

**Table 5:** Functional and Emotional Impact of Migraine

Impact	Total Population (*n* = 11,266)	Migraine Individuals With 1 TF (*n* = 961)	Migraine Individuals With ≥2 TFs (*n* = 6717)	*P*-value (≥2 TFs vs 1 TF)
Severity of pain (range from 0 to 10; mean reported)	7.4	7.3	7.5	< 0.05
Ever cancelled plans due to migraine	80% (9013)	75% (721)	83% (5575)	< 0.05
Often or always feeling frustrated by migraine	56% (6309)	55% (529)	59% (3963)	< 0.05
Migraine often or always interfering with ability to think clearly or to focus on daily life activities and tasks	52% (5838)	52% (500)	56% (3762)	< 0.05
Level of migraine interference with daily activities (“a lot” or “constantly”)	51% (5746)	48% (461)	57% (3829)	< 0.05
Often or always lacking the energy to complete daily living or felt fatigued	50% (5633)	48% (461)	54% (3627)	< 0.05
Level of impairment in daily activity due to migraine (needing to stop and rest “a lot” or “always”)	45% (5070)	42% (404)	49% (3291)	< 0.05
Often or always feeling hopeless or helpless by migraine	43% (4844)	38% (365)	47% (3157)	< 0.05

*TF* Treatment failure

### Impact on private, social, and professional life

Impact of migraine on professional, private, or social domains was reported by 87% of respondents (51% in all domains). Sixty-four percent of respondents reported that migraine had affected their private life (70% of respondents with ≥2 TFs), including relationships with friends, relatives, and partners. On average each respondent mentioned 4 negative impacts related to missing important events (birthdays, weddings) (52%), avoiding making commitments (50%), effect on sex life (49%), and feeling guilty about the impact migraine has on their family life (44%).

Social activities were also affected for 78% of respondents (82% in the ≥2 TFs subgroup). On average each respondent mentioned 3 negative impacts with the most mentioned being not participating in all activities/hobbies they used to (59%), being stopped from going to social events (57%), and being stopped from engaging in sports activities or exercise (34%).

Over the previous 3 months, 61% had relied on external support from family friends or someone else to cope with daily tasks (64% in the ≥2 TFs subgroup). In the 3 months before answering the survey, assistance was required for 12.8 days on average for physical (eg, cooking, cleaning, shopping), emotional (eg, support, comfort, understanding), and medical (eg, taking/providing medication, transport to doctor, buying medication at pharmacy) aspects of life by 86%, 66%, and 49% of respondents, respectively. In the same time period, respondents with ≥2 TFs required more support (13.6 days on average), and 54% of respondents with ≥2 TFs required help with medical aspects compared with 49% of all respondents with migraine.

### Impact of migraine on work productivity

Of all survey respondents, 70% reported that migraine has affected their professional life, which rose to 75% in the ≥2 TFs subgroup and was significantly higher than those with no TF (60%) (*P* < 0.05). The top 3 impacts of migraine on work reported by the respondents were inability to concentrate on work (52%), missing too many days of work (32%), and lack of understanding among colleagues about their condition or taking it seriously (27%). Out of the 58% of respondents who were in full- or part-time paid employment, 63% reported that their employers were aware of their migraine (68% in the ≥2 TFs subgroup), whereas only 18% reported receiving any support from their employer. Of all respondents, 9% (12% of those with ≥2 TFs) reported receiving a disability-related allowance because of their migraine.

A majority of respondents in employment (60%) reported missing ≥1 day of work in the last month due to migraine, with an average of 4.6 working days being missed in the last month, which is consistent with the average of 4.5 working days missed due to migraine (absenteeism) measured by the WPAI questionnaire. Notably, the ≥2 TFs subgroup missed an average of 5 working days due to migraine, which was significantly higher vs 3.8 days missed by those with 1 TF (*P* < 0.05). However, paid sick days reported by respondents in employment in the last month were on average 2.4 days: 2.7 days in the ≥2 TFs subgroup vs 1.7 days in those with 1 TF (*P* < 0.05).

Of the 7339 (65%) respondents who reported being currently employed, 6606 reported having worked in the last 7 days. These study participants reported that migraine led to a reduction of 13% in their working time (absenteeism) and 48% in productivity while working (presenteeism); 52% reported impairment in both overall work productivity (absenteeism and presenteeism combined) and daily activities due to migraine. Subgroup analysis according to prior treatment failure showed that impairment in all WPAI scores (absenteeism, presenteeism, and impairment in overall work productivity and daily activities) was highest in respondents with ≥2 TFs, followed by the 1 TF, no TF, and treatment-naïve subgroups.

### Healthcare resource utilization

In the previous 6 months, the majority of respondents reported visiting a general practitioner (GP) (53%), whereas smaller but notable proportions also visited a neurologist (40%), pharmacist (19%), headache specialist (15%), dentist (12%), physiotherapist (11%), and psychologist or psychiatrist (10%). A majority of respondents (58%) reported having had a brain scan (average of 2.1 scans); by comparison, 68% of respondents in the ≥2 TFs subgroup had undergone an average of 2.3 brain scans over time (Table 6), which was significantly higher than the 29% of treatment-naïve respondents who had undergone an average of 1.7 brain scans (*P* < 0.05).

**Table 6:** Resource Utilization by Number of Previously Used Migraine Preventive Treatments

	Total Population (*n* = 11,266)	Persons With Migraine With 1 TF (*n* = 961)	Persons With Migraine With ≥2 TFs (*n* = 6717)
Brain scan			
Proportion of respondents	58% (6534)	56% (538)	68% (4568)
Average number of scans	2.1	1.7	2.3
ED visits in past 12 months			
Proportion of respondents	38% (4281)	28% (269)	46% (3090)
Average number of visits	3.3	2.7	3.5
Overnight hospital stay in past 12 months			
Proportion of respondents	23% (2591)	15% (144)	29% (1948)
Average nights of stay	3.2	2.3	3.4

*ED* Emergency department; *TF* Treatment failure

In the previous 12 months, more than a third of respondents had visited the emergency department (ED) because of their migraine and had on average 3.3 visits in the last year, whereas nearly a quarter stayed in hospital overnight (3.2 nights on average) (Table 6).

## Discussion

This large worldwide study of 11,266 participants constitutes the largest survey to date conducted in individuals with migraine suffering from ≥4 MMDs and focused mostly on those who previously experienced failure of migraine prevention treatments. The size and granularity of this study have created a rich data source to describe the impact of migraine in this economically active population, with personal, social, and professional commitments and high healthcare use in unprecedented detail.

The study data reveal that individuals with migraine, especially those with a history of previous preventive treatment failures, have higher levels of unmet needs. This study describes the personal, social, humanistic, and economic burden of migraine in detail, and for the first time, it allows quantifying the burden during different phases of the migraine attack for individuals suffering from ≥4 MMDs with a history of prophylactic treatment. Although the headache phase itself caused the highest degree of impairment, the findings demonstrate that the burden of migraine extends beyond the headache phase itself and is higher for individuals who had at least one previous preventive treatment failure.

Respondents that had ≥4 MMDs reported a high rate of ED visits and overnight stays in the hospital due to migraine. Also, those with a history of failed preventive treatments for migraine had even higher utilization of healthcare resources. This trend suggests that treatment failure in migraine may be an important driver of increased healthcare utilization. These findings are consistent with the results of the IBMS study, which found that for each of the five European countries studied, chronic migraine (per ICHD-2 criteria, persons with ≥15 MMDs) was associated with additional healthcare costs attributable to an increased use of medical services and associated cost [6]. However, unlike the IBMS study, the *My Migraine Voice* participants cover a larger range of migraine individuals (≥4 MMDs), therefore providing a more comprehensive appraisal of burden and healthcare utilization.

In addition to the direct economic burden, results of this study highlight the substantial indirect economic burden imposed by migraine. Loss in work productivity and activity impairment due to migraine is higher in individuals with previously failed preventive treatments, further establishing the need for more effective treatment options in this particular population to ensure that these affected persons can fully contribute to the productive workforce and society.

The present study has some limitations for consideration. The recruitment of participants aimed to select migraine individuals with ≥4 MMDs. While the sample does not entirely mirror the overall general population of people with migraine, it allowed selecting a representative and generalizable sample of individuals with severe disease. Recruitment bias may affect the estimates where differences are present between survey participants and the population from each country. For example, individuals with 1 to 3 MMDs are not included as part of this study, nor are people with migraine without ready access to the internet or those who are not part of the catchment group contacted to participate in this survey. Pooling data from different countries may also introduce some variability in the dataset, unless country-specific nuances can be accounted for. Another limitation of this study is the use of self-reported data because diagnosis of migraine and other relevant diagnoses (eg, those used in the comorbidity index) and reporting of healthcare visits and other variables of interest cannot be clinically confirmed. However, there is no substantial reason why one would not believe a self-reported migraine diagnosis if that individual can identify what type and when the physician diagnosed him/her with migraine. The magnitude of migraine impact is likely affected by the frequency, severity, and duration of migraine, which may also be susceptible to impact from reporting and recall biases. Moreover, *My Migraine Voice* has been completed and currently no follow-ups are planned. Therefore, although it is the largest cross-sectional study specifically reporting the burden of disease in individuals with severe migraine to date, it does not benefit from the additional insights that a longitudinal study in the same population might reveal about the time evolution of migraine and its multifaceted burden.

Despite the limitations above, this study strikes a balance between the sample size needed to obtain a higher-resolution description about the burden of migraine imposed on affected individuals with the highest unmet need (≥4 MMDs) and potential caveats in generalizing its findings to all people with migraine (irrespective of disease severity) resulting from the above-mentioned limitations. The impact of migraine on the lives of affected individuals was also measured by some patient-reported outcome measures. A web-based approach appears to be an appropriate method to capture cross-sectional or longitudinal data and grants access to a population that would typically not be found in clinical settings, allowing a determination of the global burden of migraine [5, 13]. A follow-up study, IBMS-II, was conducted [14] with the objective to characterize patterns of preventive medication use in persons with migraine; it concluded that persons experiencing a higher frequency of migraine days tried more medications than those with a lower number of MMDs. *My Migraine Voice* adds to the existing literature since it includes a large number of countries, involves people with migraine meeting the ICHD-3 criteria, and assesses migraine burden during premonitory, headache, and postdrome phases of the migraine attack, thereby allowing for a comprehensive and truly global assessment of migraine burden.

## Conclusions

This study demonstrates that the burden of migraine is significant and poses several challenges among those with ≥4 MMDs and a history of prophylactic treatment failure. However, despite challenges posed by migraine, only 9% reported receiving disability allowance due to their migraine. Interestingly, the data show that individuals with migraine report some positive aspects in their migraine journey, which relate to developing a positive outlook on personal growth that is likely triggered by learning to cope with the disease. The greater resilience and strength brought on by coping with migraine suggests that if future treatments could address their existing unmet needs, these individuals with migraine will be able to maximize their contribution to society.

## Abbreviations

AMPP: American Migraine Prevalence and Prevention
CaMEO: Chronic Migraine Epidemiology and Outcomes
ED: Emergency department
GBD: Global Burden of Disease
GP: General practitioner
IBMS: International Burden of Migraine Study
ICHD-3: International Classification of Headache Disorders 3rd edition
MMD: Monthly migraine day
OBB: Online bulletin board
OTC: Over-the-counter
TF: Treatment failure
WPAI: Work Productivity and Activity Impairment
YLD: Years lived with disability
